# Evaluation of various commodities for the development of the yellow mealworm, *Tenebrio molitor*

**DOI:** 10.1038/s41598-020-67363-1

**Published:** 2020-07-08

**Authors:** Christos I. Rumbos, Ioannis T. Karapanagiotidis, Eleni Mente, Pier Psofakis, Christos G. Athanassiou

**Affiliations:** 1 Department of Agriculture Crop Production and Rural Environment, School of Agricultural Sciences, University of Thessaly, Fytokou Str., 38446 Volos, Greece; 2 Department of Ichthyology and Aquatic Environment, School of Agricultural Sciences, University of Thessaly, Fytokou Str., 38446 Volos, Greece

**Keywords:** Developmental biology, Plant sciences

## Abstract

We evaluated the suitability of forty-four commodities (i.e., cereal flours and meals, non-flour, cereal commodities, legumes and various commodities of vegetative and animal origin) as oviposition and feeding substrates for the yellow mealworm, *Tenebrio molitor*. Τen *T. molitor* adults were introduced in plastic vials containing 30 g of each commodity. At the end of the 1 week period, all adults were removed, and mortality was determined; then the vials were further incubated for additional 9 weeks. After this time, the vials were opened, and the larvae of each vial were separated from the feeding substrate, counted and weighed as a group. The efficiency of ingested food conversion was calculated for each substrate. Finally, proximate composition was calculated to determine the nutrient components of the feeding substrates tested and the *T. molitor* larvae that fed on various selected substrates. In general, adult reproduction was clearly favoured by most amylaceous substrates tested, which was in contrast to the tested legumes on which fewer offspring were produced. Similar effects were observed for larval development. Feeding on selected substrates exerted an impact on the nutrient composition of *T. molitor* larvae, with a high protein content of the substrate usually resulting in a high protein content of the larvae.

## Introduction

Over the last few years, “insect farming” has attracted considerable scientific attention, as insects are considered an alternative and sustainable nutrient source for animal feed and human food^1–5^. Among the most promising insect species for industrial utilization and commercial large-scale production is the yellow mealworm, *Tenebrio molitor* L. (Coleoptera: Tenebrionidae). This species is one of the largest stored-product beetles (adult body length between 12 and 20 mm) that is commonly found infesting stored agricultural products^6^. It is a cosmopolitan stored product insect pest that is found in various types of facilities and commodities, mainly grains and related amylaceous commodities, such as flour, bran and pasta^6^. Nevertheless, there is strong interest in its utilization as a food source for humans and animals, including fish^1^. This is because *T. molitor* larvae are highly nutritious with high protein and lipid contents^7,8^. Because of their nutritional value, the larvae of *T. molitor* are commonly used as feed for pets (e.g., reptiles and birds)^9^, whereas they have been successfully evaluated as a feed ingredient in pig^10^ and poultry diets^11,12^, as well as in artificial diets for the mass-rearing of beneficial organisms, such as the predatory lady beetle, *Coleomegilla maculata* De Geer (Coleoptera: Coccinellidae)^13^. Recently, *T. molitor* was included in the list of insect species that are allowed to be used as ingredients in fish feeds in the EU^14^, whereas its exploitation for human consumption has gained increasing traction among consumers in Western countries^15^. This recent development considerably strengthened the “insect farming” industry in Europe that is expected to grow further in future years^16^.

The development of diets capable of supporting and, ideally, maximizing insect growth and development has been identified as one of the major challenges for the insect-producing industry in terms of achieving efficient, cost-effective and sustainable insect production for food and feed^17,18^. Insect diets should be species-specific to meet the nutritional requirements of the different insect species and be specific to the different life stages to maximize the total larval biomass production and increase adult reproductive performance^17^. Initial research on the nutritional requirements of *T. molitor* dates back to the 1950s, when the nutritional needs of its larvae were studied and diets consisting of 80–85% carbohydrates were proposed^19^, whereas advanced detailed work was conducted in subsequent decades^20–26^. To date, artificial diets of *T. molitor* in commercial insect farms have been primarily wheat bran-based; however, they are not nutritionally designed specifically for *T. molitor*, as occurs for other livestock animals (e.g., poultry and farmed fish)^18^. Instead, compound animal feeds, originally designed for other traditional farm animals, have been commonly used for *T. molitor* production^18^.

Several previous studies have evaluated various diets for the mass production of *T. molitor*. For instance, dry potato flour, dry egg white, soy protein, peanut oil, canola oil and salmon oil were evaluated as nutritional supplements to the *T. molitor* diet, and in comparison to a carbohydrate-rich diet, a diet with an increased content of proteins and lipids significantly enhanced most biological parameters measured^9^. Based on these results, a diet consisting of 80% wheat bran and 20% of a supplement composed of 17:2:1 parts of dry potato, dry egg white and soy protein, respectively, was designed and used to study the effect of larval density on the food utilization efficiency of *T. molitor*^27^. A similar diet, consisting of 80% wheat bran and 20% of a supplement composed by dry potato, dry egg white, soy protein and peanut oil in 80:10:5:5 proportion, was used to morphometrically analyse *T. molitor* instars^28^. In another study on the *T. molitor* instar number and development time under different diet regimes, wheat bran-based diets supplemented with dry potato and egg white in various proportions were evaluated^29^. However, the information available to date is not exhaustive; moreover, data on the effect of individual feeding substrates that could serve as *T. molitor* diet components are limited.

Data on the growth and performance of *T. molitor* on single-component diets could improve our knowledge for *T. molitor* nutrition and contribute to the development of nutritionally balanced dietary mixtures for the mass production of this species. This is particularly important, as this species has an extremely wide range of food preferences, so finding a mixture of suitable components is more demanding than it is for oligophagous stored product-insects. In a catalogue of commodities that are associated with stored-product insects, *T. molitor* was associated with 51 different types of stored commodities^6^. Nevertheless, to our knowledge, a comparative study evaluating a broad range of various feeding substrates that could be included in a mass production diet for *T. molitor* is missing. In this framework, the objective of the present study was to generate baseline information on the suitability of a broad spectrum of feeding substrates for *T. molitor* adult reproduction and larval development. Therefore, forty-four feeding substrates of vegetative and animal origin were evaluated individually as oviposition and larval development substrates for *T. molitor*. Finally, the effect of the selected feeding substrates on the nutrient composition of *T. molitor* larvae was investigated.

## Results

### Nutrient composition of feeding substrates and *T. molitor* larvae

The protein content varied considerably among the feeding substrates tested (0.4–72%) (Table 1). All cereals had a protein content lower than 15% (as fed), with the exception of corn gluten, whereas the respective values for legume flours and oil plants ranged between 22 and 42%. The gross energy of the substrates tested ranged between 14.1 and 27.2 kJ/g. The protein content of *T. molitor* larvae that fed on the different substrates tested ranged between 41 and 68%, whereas the lipid content fluctuated between 14 and 38% (Table 2). Linear regression analysis showed a weak correlation of the substrate protein content with the protein content of the larvae (R^2^ = 0.36, F = 20.7, P < 0.001).

**Table 1:** Moisture (% as fed), crude protein (% as fed) and gross energy (kJ/g) of the commodities tested in the trials.

Substrate	Moisture (%)	Protein (%)	Energy (kJ/g)	Substrate	Moisture (%)	Protein (%)	Energy (kJ/g)
Cereal flours				Legume flours			
Barley meal	10.7	9.6	16.4	Bean flour	11.9	23.4	16.5
Corn flour	11.8	6.8	15.2	Chickpea flour	9.3	22.9	17.6
Durum wheat flour	15.9	12.7	16.3	Fava flour	10.2	28.7	16.7
Millet flour	10.8	7.4	17.3	Lentil flour	11.3	29.0	16.7
Oat flour	9.8	8.5	17.1	Lupine meal	7.5	34.8	19.5
Rice flour	12.0	7.9	15.5	Soya meal	10.6	42.4	16.9
Rye flour	12.7	7.4	15.7	Various substrates			
White flour	11.6	11.5	15.9	Amaranth	11.0	15.2	17.6
Whole meal flour	11.3	11.3	15.8	Beet pulp meal	7.4	9.3	15.7
Zea flour	11.6	11.1	16.0	Buckwheat flakes	11.9	17.2	16.6
Other cereal substrates				Buckwheat flour	13.7	12.2	16.0
Barley flakes	11.8	9.7	16.1	Coconut flour	6.3	21.2	17.8
Corn gluten	6.6	72.7	21.8	Cotton seed meal	7.5	23.5	20.0
Corn starch	11.9	0.4	15.2	Egg-layer hen feed	10.6	16.3	15.3
Millet flakes	12.7	12.4	16.5	Leen flour	8.1	32.2	18.8
Millet grains	11.8	11.6	16.7	Milk-based feed	9.9	36.7	16.9
Oat bran	10.5	14.6	18.0	Milk powder	9.2	21.0	21.1
Oat flakes	10.5	12.7	17.6	Potato dry white	16.7	0.4	14.1
Rye flakes	11.2	6.5	15.9	Quinoa flakes	11.1	10.6	17.2
Semolina fine	11.0	10.6	16.0	Quinoa flour	7.2	14.6	17.8
Semolina coarse	11.3	11.2	16.1	Rapeseed meal	10.2	30.6	17.9
Vittes (wheat byproduct)	10.9	11.2	15.9	Sunflower meal	7.2	26.2	16.9
Wheat bran	12.7	14.2	17.0	Whole egg powder	3.7	46.5	27.2

**Table 2:** Proximate composition (% on dry weight basis) (moisture, protein and lipid content) of *Tenebrio molitor* larvae fed with various substrates.

Substrate	Moisture (%)	Protein (%)	Lipids (%)
Milk-based feed	62.9 ± 0.5 ab	68.1 ± 0.3 a	14.1 ± 0.4 c
Wheat bran	63.9 ± 1.6 a	63.3 ± 1.8 ab	19.3 ± 1.4 bc
Chickpea flour	61.8 ± 4.4 ab	62.9 ± 0.1 abc	n.d.
Wheat by-product	60.0 ± 0.8 ab	59.6 ± 1.2 bcd	n.d.
Millet grains	61.5 ± 2.2 ab	58.9 ± 0.3 bcde	n.d.
Buckwheat flour	62.7 ± 0.7 ab	58.2 ± 1.9 bcdef	n.d.
Whole meal flour	62.1 ± 0.6 ab	56.1 ± 0.8 cdef	n.d.
Egg-layer hen feed	58.1 ± 1.0 b	55.2 ± 2.2 def	23.6 ± 3.0 b
Rye flakes	66.6 ± 2.1 a	55.0 ± 0.5 def	n.d.
Semolina (fine)	63.1 ± 0.5 ab	53.5 ± 0.1 def	n.d.
Semolina (coarse)	61.7 ± 0.5 ab	53.0 ± 1.2 def	n.d.
Barley meal (for feed)	62.3 ± 1.0 ab	52.7 ± 1.2 def	n.d.
White flour	62.5 ± 1.8 ab	52.2 ± 1.3 ef	n.d.
Zea flour	61.2 ± 0.6 ab	52.0 ± 0.8 f	n.d.
Durum wheat flour	60.0 ± 0.4 ab	41.4 ± 0.4 g	38.8 ± 1.4 a
F	2.2	29.4	18.1
*P*	0.022	< 0.001	< 0.001

Within each column, means followed by the same lowercase letter are not significantly different (for moisture and protein content df = 14, 44, whereas for lipid content df = 3, 11; Tukey–Kramer HSD test at P = 0.05).

*n.d.* not determined.

### Cereal flours

The mortality of *T. molitor* adults after 1 week in vials with cereal flours was generally low (< 10%), with the exception of those in oat flour (Table 3). Progeny production varied significantly among the cereal flours tested, with the highest values for oat and rye flour. The highest total larval weight on a dry-matter basis was recorded in durum wheat flour, zea flour and white flour, which was significantly higher than that recorded in rice flour and millet flour. Likewise, the highest mean individual larval weight was recorded for durum wheat flour and white flour. Similar results were obtained for the total dry feed consumed. Finally, significant differences were recorded among the efficiency of ingested feed conversion (ECI) values that were calculated for the various cereal flours. The highest ECI value was noted for durum wheat flour and was significantly higher than that of all other cereal flours tested, with the exception of zea flour.

**Table 3:** Adult mortality (%) (after 1-week), number of alive larvae, total larval dry weight (mg), mean individual larval dry weight, total dry feed consumed (mg), and efficiency of ingested food conversion (ECI, %) of *Tenebrio molitor* larvae fed on different cereal flours for 10 weeks.

Diet components	Adult mortality (%)	Number of alive larvae	Total larval dry weight (mg)	Individual larval dry weight (mg)	Total dry feed consumed (mg)	ECI (%)
Barley meal	3.3 ± 2.1 ab	52.0 ± 7.2 b	170.6 ± 39.5 abc	3.3 ± 0.4 ab	2,064.7 ± 251.1 abc	8.0 ± 1.0 bc
Corn flour	6.7 ± 3.3 ab	120.7 ± 19.9 ab	67.8 ± 7.6 cd	0.6 ± 0.1 ef	1,376.4 ± 223.7 cd	5.2 ± 0.4 bcd
Durum wheat flour	6.7 ± 3.3 ab	123.0 ± 20.6 ab	517.2 ± 116.0 a	4.3 ± 0.9 a	3,313.3 ± 890.6 abc	16.9 ± 1.4 a
Millet flour	1.7 ± 1.7 b	49.5 ± 13.1 b	15.3 ± 3.9 e	0.3 ± 0.1 f	878.6 ± 208.8 d	1.8 ± 0.3 e
Oat flour	16.7 ± 4.9 a	153.5 ± 5.4 a	104.2 ± 7.7 bcd	0.7 ± 0.1 ef	1,881.2 ± 144.1 abc	5.6 ± 0.2 bcd
Rice flour	1.7 ± 1.7 b	51.5 ± 6.4 b	50.3 ± 16.1 de	1.0 ± 0.3 def	1,519.1 ± 137.5 bcd	3.1 ± 0.7 de
Rye flour	6.7 ± 3.3 ab	149.3 ± 13.4 a	148.1 ± 18.2 abc	1.0 ± 0.1 cde	2,986.7 ± 212.6 ab	4.9 ± 0.4 bcd
White flour	3.3 ± 2.1 ab	107.5 ± 37.1 ab	251.6 ± 112.5 abc	2.1 ± 0.5 bc	3,508.1 ± 726.5 a	5.6 ± 1.7 cd
Whole meal flour	0.0 ± 0.0 b	56.2 ± 19.9 b	99.6 ± 34.4 cd	1.8 ± 0.2 bcd	1,891.4 ± 318.7 abcd	4.6 ± 0.9 cd
Zea flour	8.3 ± 4.8 ab	121.3 ± 18.9 ab	293.1 ± 42.3 ab	2.5 ± 0.1 b	3,118.2 ± 300.9 ab	9.2 ± 0.6 ab
F	2.4	5.7	14.9	22.9	7.2	18.5
*P*	0.023	< 0.001	< 0.001	< 0.001	< 0.001	< 0.001

In all cases, values represent means ± SEM (n = 6).

Within each column, means followed by the same lowercase letter are not significantly different (in all cases df = 9, 59; Tukey–Kramer HSD test at P = 0.05).

### Non-flour, cereal substrates

Adult mortality in various non-flour, cereal substrates did not exceed 7% (Table 4). No progeny production was recorded in corn gluten (data not presented). Most larvae were produced in wheat bran, followed by wheat byproduct (vittes), semolina (fine) and corn starch. Progeny production was low in millet grains, rye flakes and barley flakes, for which the number of larvae did not exceed 53 larvae/vial. The total larval biomass produced was highest when *T. molitor* larvae were reared in wheat bran, and this biomass amount was significantly higher than that on all other substrates tested in this series of bioassays. A significant substrate effect on larval growth was also observed when the mean individual larval weight was calculated. In this case, the highest mean individual larval weights were calculated for rye flakes and wheat bran and were significantly higher than the remaining substrates tested. Specifically for the rye flakes, relatively few but large larvae were produced. Finally, the highest ECI value, as well as total dry feed consumed, was estimated for wheat bran.

**Table 4:** Adult mortality (%) (after 1-week), number of alive larvae, total larval dry weight (mg), mean individual larval dry weight, total dry feed consumed (mg), and efficiency of ingested food conversion (ECI, %) of *Tenebrio molitor* larvae fed on various non-flour, cereal substrates for 10 weeks.

Diet components	Adult mortality (%)	Number of alive larvae	Total larval dry weight (mg)	Individual larval dry weight (mg)	Total dry feed consumed (mg)	ECI (%)
Barley flakes	3.3 ± 2.1	7.2 ± 2.6 e	20.5 ± 3.6 f	4.7 ± 1.1 b	721.3 ± 132.9 ef	3.4 ± 0.8 e
Corn starch	3.3 ± 2.1	103.8 ± 24.9 abc	39.3 ± 9.1 ef	0.4 ± 0.1 f	1,362.8 ± 127.3 cde	3.0 ± 0.7 e
Millet flakes	3.3 ± 2.1	102.7 ± 7.9 abc	64.3 ± 11.2 de	0.6 ± 0.1 ef	1,140.7 ± 92.4 def	5.5 ± 0.6 d
Millet grains	0.0 ± 0.0	53.0 ± 8.5 c	198.9 ± 25.7 bc	3.9 ± 0.2 b	1,359.0 ± 194.8 cde	14.8 ± 0.3 a
Oat bran	3.3 ± 2.1	70.3 ± 9.8 bc	56.6 ± 7.1 de	0.8 ± 0.1 def	573.2 ± 69.7 f	9.9 ± 0.3 abc
Oat flakes	0.0 ± 0.0	69.8 ± 11.9 bc	136.5 ± 50.7 cde	1.6 ± 0.4 cde	1,742.4 ± 561.5 cde	6.8 ± 0.8 cd
Rye flakes	3.3 ± 0.0	20.7 ± 5.2 d	146.8 ± 23.6 bcd	8.3 ± 1.1 a	1,247.1 ± 155.7 cde	11.6 ± 0.5 ab
Semolina (coarse)	1.7 ± 1.7	95.5 ± 14.6 abc	292.7 ± 48.3 b	3.0 ± 0.1 bc	2,559.8 ± 313.3 bc	11.1 ± 0.6 ab
Semolina (fine)	0.0 ± 0.0	103.3 ± 22.9 abc	128.0 ± 27.1 bcd	1.3 ± 0.1 def	1,597.0 ± 264.5 cd	7.7 ± 0.6 bcd
Vittes (wheat byproduct)	5.0 ± 2.2	129.3 ± 12.4 ab	237.3 ± 23.1 b	1.9 ± 0.2 cd	3,353.0 ± 152.8 b	7.0 ± 0.4 bcd
Wheat bran	6.7 ± 2.6	183.8 ± 11.3 a	1,431.7 ± 114.3 a	8.2 ± 0.9 a	9,583.6 ± 748.5 a	14.9 ± 0.4 a
F	1.2	28.2	43.5	40.0	36.1	30.3
*P*	0.281	< 0.001	< 0.001	< 0.001	< 0.001	< 0.001

In all cases, values represent means ± SEM (n = 6).

Within each column, means followed by the same lowercase letter are not significantly different (in all cases df = 10, 65; Tukey–Kramer HSD test at P = 0.05. Where no letters exist, no significant differences were noted).

### Legume flours and meals

Adult mortality increased compared to that in the previous two series of bioassays and reached 18.3, 23.3 and 30.0% in fava flour, lupine meal and lentil flour, respectively (Table 5). Progeny production in legume flours and meals decreased compared to the number of larvae produced in cereal substrates and just reached 81 and 90 larvae/vial in fava and chickpea flour, respectively. Total larval weight was the highest in chickpea and lentil flour and the lowest in the bean flour and soya meal. Similarly, ECI got its highest values for lentil and chickpea flour.

**Table 5:** Adult mortality (%) (after 1-week), number of alive larvae, total larval dry weight (mg), mean individual larval dry weight, total dry feed consumed (mg), and efficiency of ingested food conversion (ECI, %) of *Tenebrio molitor* larvae fed on different legume flours for 10 weeks.

Diet components	Adult mortality (%)	Number of alive larvae	Total larval dry weight (mg)	Individual larval dry weight (mg)	Total dry feed consumed (mg)	ECI (%)
Bean flour	16.7 ± 3.3 ab	24.8 ± 3.3 bc	22.4 ± 4.5 b	0.9 ± 0.1 bcd	1,047.5 ± 55.9 b	2.1 ± 0.4 c
Chickpea flour	15.0 ± 4.3 ab	90.5 ± 21.8 a	97.3 ± 23.8 a	1.1 ± 0.1 bc	1,310.0 ± 255.4 b	7.1 ± 0.5 a
Fava flour	18.3 ± 4.0 ab	81.7 ± 20.2 a	46.8 ± 9.1 ab	0.6 ± 0.1 cd	943.7 ± 108.3 b	4.9 ± 0.7 ab
Lentil flour	30.0 ± 8.2 a	71.7 ± 10.3 a	90.6 ± 7.1 a	1.4 ± 0.1 b	1,061.5 ± 59.5 b	8.6 ± 0.7 a
Lupine meal	23.3 ± 6.1 ab	16.5 ± 2.4 c	46.2 ± 10.2 ab	2.7 ± 0.5 a	2,033.1 ± 48.2 a	2.3 ± 0.5 c
Soya meal	6.7 ± 3.3 b	48.5 ± 9.9 ab	27.2 ± 4.4 b	0.6 ± 0.1 d	890.3 ± 23.9 b	2.5 ± 0.5 bc
F	2.3	11.0	7.2	14.7	8.7	15.9
*P*	0.048	< 0.001	< 0.001	< 0.001	< 0.001	< 0.001

In all cases, values represent means ± SEM (n = 6).

Within each column, means followed by the same lowercase letter are not significantly different (in all cases df = 5, 29; Tukey–Kramer HSD test at P = 0.05).

### Various substrates (vegetative or animal origin)

High adult mortality was observed in the milk-based feed and in quinoa flour, whereas in the remaining substrates tested, it did not exceed 13% (Table 6). No progeny production was recorded in amaranth, beet pulp meal, sunflower meal, cotton seed meal and leen flour (data not presented), while in coconut flour, dead early-instar *T. molitor* larvae were present during the evaluation. Despite the high adult mortality noted in the milk-based feed, progeny production in this substrate was high and significantly higher than that in the remaining substrates evaluated, with the exception of buckwheat flour and egg-layer hen feed. Furthermore, in this series of bioassays, the best growth parameters were noted for egg-layer hen feed, for which the total, as well as the individual larval weight, was significantly higher than the respective values for all the remaining substrates. Finally, high ECI values were calculated, apart from the egg-layer hen feed, for the milk-based feed and buckwheat flour.

**Table 6:** Adult mortality (%) (after 1-week), number of alive larvae, total larval dry weight (mg), mean individual larval dry weight, total dry feed consumed (mg), and efficiency of ingested food conversion (ECI, %) of *Tenebrio molitor* larvae fed on various substrates of vegetative or animal origin for 10 weeks.

Diet components	Adult mortality (%)	Number of alive larvae	Total larval dry weight (mg)	Individual larval dry weight (mg)	Total dry feed consumed (mg)	ECI (%)
Buckwheat flakes	3.3 ± 3.3 b	21.3 ± 1.4 e	33.4 ± 10.1 ef	1.5 ± 0.4 cd	541.0 ± 92.4 de	6.0 ± 0.9 bc
Buckwheat flour	10.0 ± 2.6 b	155.0 ± 15.1 ab	220.4 ± 32.2 c	1.4 ± 0.1 cd	2,486.5 ± 220.8 bc	8.7 ± 0.6 ab
Dry potato powder	6.7 ± 3.3 b	24.0 ± 2.3 de	14.4 ± 1.1 f	0.6 ± 0.1 de	1,662.6 ± 32.8 bc	0.9 ± 0.1 d
Egg-layer hen feed	6.7 ± 3.3 b	149.2 ± 25.3 ab	1744.7 ± 432.6 a	11.1 ± 1.9 a	10,803.6 ± 2,411.9 a	15.5 ± 0.7 a
Egg whole powder	13.3 ± 4.2 b	4.7 ± 1.2 f	3.8 ± 1.0 g	0.8 ± 0.1 de	554.7 ± 163.1 e	1.9 ± 1.1 d
Milk-based feed	48.3 ± 13.5 a	180.0 ± 24.9 a	505.7 ± 34.5 b	3.1 ± 0.5 b	3,624.2 ± 245.4 ab	14.0 ± 0.5 ab
Milk powder	5.0 ± 3.4 b	28.0 ± 6.9 de	13.9 ± 3.5	0.5 ± 0.1 e	572.1 ± 113.2 de	7.1 ± 5.5 cd
Quinoa flakes	3.3 ± 3.3 b	56.0 ± 8.1 c	52.9 ± 6.7 de	1.0 ± 0.2 cde	915.9 ± 98.1 cde	5.9 ± 0.8 bc
Quinoa flour	24.0 ± 4.0 ab	83.8 ± 17.6 bc	119.3 ± 18.4 cd	1.5 ± 0.1 cd	1,561.8 ± 277.8 bcd	7.8 ± 0.5 abc
Rapeseed meal	1.7 ± 1.7 b	50.2 ± 7.0 cd	80.0 ± 7.5 d	1.7 ± 0.1 bc	1,211.6 ± 63.0 bcd	6.5 ± 0.3 abc
F	7.1	49.1	102.9	49.4	18.2	16.1
*P*	< 0.001	< 0.001	< 0.001	< 0.001	< 0.001	< 0.001

In all cases, values represent means ± SEM (n = 6).

Within each column, means followed by the same lowercase letter are not significantly different (in all cases df = 9, 59; Tukey–Kramer HSD test at P = 0.05. Where no letters exist, no significant differences were noted).

Linear regression analysis showed no significant correlation between the protein content of the substrates and the total (R^2^ = 0.03), individual larval weight (R^2^ = 0.01) and the ECI (R^2^ = 0.01), as well as between the energy of the substrates and the total (R^2^ = 0.15), individual larval weight (R^2^ = 0.02) and the ECI (R^2^ = 0.06).

## Discussion

Diet plays an important role for insect growth and performance; therefore, the development of an effective artificial diet has been identified as a crucial component of insect-producing systems, such as the mass production of beneficial arthropods in general^30^, phytophagous insects^31,32^ or insects reared as a source of nutrients for food or feed^33,34^. In the case of *T. molitor*, considerable effort has been focused on the development of artificial diets that would maximize the biomass production of highly nutritious larvae, with a concomitant reduction in their developmental time. For instance, significant differences were reported in the feed conversion efficiency, survival and developmental time of *T. molitor* larvae that were fed four diets composed of various food byproducts^35^. A detailed review of the diets and protein sources that have been evaluated to date for the rearing of *T. molitor* was recently published^36^. Our results build on previous studies showing that feed composition has a major impact on *T. molitor* growth performance. In our study, the highest amount of total larval biomass was produced in specific amylaceous commodities, such as wheat bran, durum wheat flour, zea flour and white flour, as well as in the two compound feeds tested (egg-layer hen feed and milk-based feed). In contrast, low values were recorded for total larval weight produced in the different legume flours tested. In terms of individual larval weight, high values were recorded for the aforementioned feeding substrates, as well as for other substrates, such as rye and barley flakes, millet grains and lupine meal. Similar patterns were observed for the ECI values and the total dry feed consumed. Indicatively, the ECI was 14% and 15.5% for the milk-based and the egg-layer hen feed, respectively, whereas it was between 7 and 8% for substrates such as semolina and chickpea flour. For wheat bran, a commonly used feeding substrate for *T. molitor*, the calculated ECI value (14.9%) was similar to the respective values reported in previous studies^9^. In a similar study, ECI values were in the range of 10–12% for diets with high protein content (approximately 22% protein), whereas they did not exceed 8% in low-protein content diets (approximately 13% protein)^35^. In the same study, feed conversion efficiencies were calculated between 3.9 and 19.1% for *T. molitor* larvae fed low and high protein diets, indicating that the quality of the diet has a large impact on the food utilization parameters. Similarly, feed conversion rates (weight ingested food/weight gained) ranging from 3.4 to 6.1 were calculated for different quality diets^37^. In our case, we did not test rearing diets in terms of mixtures of different components but only single feeding substrates that could potentially be used as suitable dietary components. Nevertheless, some of the substrates examined here, such as specific amylaceous commodities, can be evaluated further as major dietary components for mass production of *T. molitor* as diets that contain a mixture of various feedstuffs could potentially be relatively more nutritionally balanced, thus fulfilling the nutritional requirements of insects. For instance, larvae of the confused flour beetle, *Tribolium confusum* Jacquelin du Val (Coleoptera: Tenebrionidae), that were fed a three-component diet mix grew faster compared to larvae fed on each of the individual components^38^. In general, insects typically grow faster with high-protein content diets^17^. In our study, however, the growth of *T. molitor* larvae was restricted in most legume flours tested, although of the substrates, these flours had the highest protein content (22.9–42.4%). For instance, in soya meal, which is an extremely rich protein source (42.4% protein), the ECI was only 2.5% and the total dry feed consumed did not exceed 891 mg. This result was consistent with what was previously noted; i.e., soybean contains a potent trypsin inhibitor that could negatively influence larval growth^39^. Knowledge on the nutrition of insects is still in its infancy, but in the nutrition of other farm animals, it is known that an excess dietary protein level, above the amino acid requirements for maximum growth, could result in a reduced growth rate due to the energy used to metabolize excess absorbed amino acids^40^. In addition, the restricted growth of *T. molitor* larvae feeding on legumes could also be attributed to a much lower digestibility compared to that for amylaceous commodities. In this context, it becomes evident that protein content in a given commodity, though an important variable, should not be considered as the only reliable indicator for the suitability of this commodity for *T. molitor* population growth.

Insect adult fertility and reproduction can also be affected by diet composition. In general, higher reproductive rates have been recorded for females fed a diet with a high protein content^17^. For instance, higher reproductive rates, expressed as number of offspring per female per day, were reported for *T. molitor* females fed diets amended with soy protein compared to that with wheat bran and dry potato powder^9^. In a similar work, significantly more eggs (11 eggs) were produced daily with a protein enriched diet compared to a low-protein diet (25 and 10% total protein content, respectively)^41^. However, only a few studies on the effect of diet on the reproductive rates of *T. molitor* are available, as more studies mainly focus on the dietary impact on growth parameters. In our study, female fecundity, expressed as the total number of offspring, was significantly affected by the feeding substrate. Most larvae were produced in the substrates that also gave the best results in terms of growth, namely wheat bran, milk-based and egg-layer hen feed, and in buckwheat and rye flour. In contrast, fewer larvae were present in the legume flours tested, although their protein content was higher compared to that of wheat bran and the remaining amylaceous flours evaluated. Nevertheless, at least for some commodities, progeny production counts may not be related to female fecundity but to increased larval mortality after egg hatching. It also seems that the form of a specific feed substrate may also have an effect on the growth and reproductive performance of *T. molitor*. For instance, although millet flakes and millet grains have a similar nutrient profile, millet flakes favoured adult reproduction, whereas enhanced larval growth was recorded in millet grains.

Insect nutrient composition is affected by various factors, such as life stage and environmental conditions (e.g., temperature and relative humidity); however, diet seems to play a major role^42,43^. Therefore, many researchers have proposed that diet can be used as a tool for the manipulation, to a certain extent, of the body composition of insects and therefore their nutritional value to meet different nutritional demands for various uses^35,37,43–46^. This scenario can be achieved either by gut loading, i.e., by providing a diet with increased concentration of a specific nutrient for a short period of time before insect harvesting and consequently increasing the concentration of this nutrient in the insect digestive tract^43–46^, or by alterations to the body composition of the insect per se after providing a specific diet over the long term^35,37^. For instance, the chemical composition of penultimate instars and adults of the migratory locust, *Locusta migratoria* L. (Orthoptera: Acrididae), that were fed throughout their lives (hatchlings to penultimate instars and adults) three different diets varied considerably, enabling its manipulation through the diet^47^. Similarly, the Ca content of *T. molitor* larvae increased when fed high-Ca diets for short periods, enhancing their nutritional value for bone mineralization of growing chickens^46^. In our study, *T. molitor* larvae were starved prior to harvesting; therefore, we can assume that the impact of the content of the larval digestive tract on the results of the nutrient composition analysis of the larvae was minimal; i.e., the results of the proximate composition reflect the nutrient content of the insect itself. In general, the effect of diet on the protein content of *T. molitor* larvae over various studies has been considered to be minimal, whereas diet has been believed to exert a significant influence on the lipid content^37,48^.

However, there are cases where the high protein content of the feed resulted in a high dry-matter protein content of *T. molitor* larvae^18^. Similarly, our results showed that feeding *T. molitor* larvae the high-in-protein milk-based feed or chickpea flour resulted in a high protein content of the larvae. Apart from the diet, insect nutrient composition can be affected by life stage and size^42^. For instance, increased fat content was recorded in giant, juvenile hormone-treated *T. molitor* larvae compared to that of regular larvae after 3 days of gut loading^49^. In our study, there were substantial differences in the sizes of the larvae, which were analyzed for nutrients, with mean individual weights ranging between 1.1 and 11.1 mg. However, linear regression analysis showed no significant correlation of larval size with larval protein content (R^2^ = 0.01).

Apart from the profile of each of the substrates tested, our population growth experiments also contained a water source, i.e., potato slices that provided the moisture that is essential for *T. molitor* rearing. Hence, although this species is associated, as a stored-product insect, with dry commodities, the addition of a water source is essential to increase its fecundity and longevity. Although the data are not presented here, our preliminary tests clearly suggested that the absence of a water source resulted in high adult mortality, and reduced progeny production capacity. Interestingly, *T. molitor* is one of the few stored-product insect species that can easily absorb moisture from the air, through their rectal complex^50^, which makes larvae able to compensate for water loss. This is why *T. molitor* larvae are not substantially affected by desiccation caused by diatomaceous earths^51^. Nevertheless, our data clearly demonstrate that the population growth of this species is notably supported by the addition of a water source.

In conclusion, our results shed light on the suitability of a broad spectrum of commodities as oviposition and feeding substrates for *T. molitor*. Adult reproduction was clearly favoured by most amylaceous substrates tested, in contrast to legumes on which fewer offspring were produced. Similar effects were observed for larval development, which can be considered a direct consequence of adult longevity. Finally, feeding on selected substrates impacted the nutrient composition of *T. molitor* larvae, an effect that needs further investigation. At a time when the insect industry is attempting to produce the necessary quantities to cover current and future market demands for insects, the results of the present study contribute to the development of diets and dietary formulations that will allow fast larval growth and development.

## Materials and methods

### Colony maintenance

The *T. molitor* colony used was established in 2016 from a stock colony provided by the Benaki Phytopathological Institute (Attica, Greece), and since then has been continuously grown in the Laboratory of Entomology and Agricultural Zoology of the University of Thessaly. *Tenebrio molitor* was reared on plastic trays (25 × 35 × 10 cm) with a fine mesh nylon screen on the lid. Approximately 400 adults were placed in oviposition trays with 1 kg of wheat bran as food source and oviposition substrate. After 1 week, adults were removed and transferred to new substrate, whereas newly hatched larvae remained in the tray for a period of approximately 4 months, during which food (wheat bran) was refilled as needed. Adults and larvae were provided with slices of fresh potatoes twice a week. The colony was kept at 26 °C, 50% relative humidity and continuous darkness. Adults, < 1-month-old were used for the bioassays.

### Experimental setup

The method used is a modification of the one described for the evaluation of the population growth of the khapra beetle, *Trogoderma granarium* Everts (Coleoptera: Dermestidae), on different commodities^52^. Plastic cylindrical vials (7.5 cm in diameter, 8.8 cm in height) were used as the experimental units for the trials. Each vial was filled with 30 g of each feeding substrate, using different vials for each substrate. In a first series of bioassays, ten cereal flours and meals (i.e., barley meal, corn flour, durum wheat flour, millet flour, oat flour, rice flour, rye flour, white flour, whole meal flour and zea flour) were evaluated as oviposition and feeding substrates for *T. molitor* adults and larvae, respectively. In another series of bioassays, the following non-flour, cereal commodities were tested: barley flakes, oat flakes, millet flakes, rye flakes, oat bran, wheat bran, corn starch, corn gluten, millet grains, semolina (fine and coarse) and vittes (wheat byproduct). Finally, in two more series of bioassays, legume commodities (i.e., chickpea flour, fava flour, lentil flour, bean flour, soya meal and lupine meal), as well as various commodities of vegetative and animal origin (i.e., leen flour, cotton seed meal, cotton cake, beet pulp meal and sunflower meal, coconut flour, egg-layer hen feed, milk-based feed, milk powder, rapeseed meal, buckwheat flakes, buckwheat flour, dry potato powder, quinoa flakes, quinoa flour, as well as egg whole powder) were evaluated. All feed substrates were purchased from the local market. For the bioassays, ten mixed-sex adults of *T. molitor* were introduced in each vial and left to oviposit for 1 week. In all treatments, adults were provided with a slice of fresh potato at the beginning of the trial. After 1 week, adults, together with the potato slides, were removed and adult mortality was determined. For all bioassays, vials remained for additional 9 weeks at 26 °C, 50% relative humidity and continuous darkness. After this interval, the vials were opened and the larvae of each vial were separated from the feeding substrate, counted and weighed as a group to calculate the total larval fresh weight produced. Furthermore, the total larval dry weight for each group was determined by proximate analysis of the moisture content of the larvae (see “Analysis of nutrient composition”). To calculate the mean individual larval dry weight, the total larval dry weight was divided by the total number of larvae produced. To calculate the amount of feed consumed (FC), the remaining feed was weighed and subtracted from the total amount of the feed provided. As a feed utilization parameter, the efficiency of ingested feed conversion (ECI, %), expressed on dry matter, was calculated as: ECI (%) = DWG × 100/FC, where DWG was the total larvae weight gained on dry matter, considering zero values as the initial larvae weight^53^.

### Analysis of nutrient composition

At the end of the feeding experiments, the larvae were harvested, left starved for 24 h and then killed by freezing at − 20 °C and further stored at this temperature until analysis. Proximate nutrient composition was carried out to the tested feeding substrates (moisture, protein content, gross energy) and the *T. molitor* larvae (moisture, protein and lipid content) fed with various selected substrates. Briefly, samples were dried to constant weight in an oven at 105 °C for 24 h to determine their moisture content, and were further grounded to a fine size by the use of a stainless-steel mill (Thermomix TM31-1, Vorwerk Elektrowerke GmbH & Co. KG, Wuppertal, Germany). The crude protein content of the feeding substrates and *T. molitor* larvae was determined by Kjeldahl analyses (N × 6.25; behr Labor-Technik GmbH, Germany). The crude fat of *T. molitor* larvae was determined by exhaustive Soxhlet extraction using petroleum ether (40–60 °C, BP) using a Soxtherm Multistat/SX PC (Sox-416 Macro, Gerhard, Germany). Finally, gross energy content of the feeding substrates tested was determined adiabatically using an IKA^®^ oxygen bomb calorimeter (C5000; IKA Werke GmbH, Staufen, Germany).

### Statistical analysis

Prior to analysis, data were checked for normality using Shapiro–Wilk’s test^54^. The percentage of adult mortality, the number of progeny (alive larvae), the total and individual dry larval weight, the total dry feed consumed and the ECI values were non-normally distributed, thus, data were log (x + 1) transformed and further checked for normality. Afterwards, all data were submitted to a one-way ANOVA, with the feeding substrate as main effect. When significant differences were found among treatments, the Tukey–Kramer HSD test was used to statistically compare the values obtained for the different feeding substrates^54^. The same procedure was also followed for the proximate composition of the *T. molitor* larvae fed on various substrates. Additionally, linear regression analysis was performed to assess possible correlations between the protein content and the energy of the tested substrates, the total and individual dry larval weight and the ECI. The same process was also followed to check if there is any significant correlation between the protein content of the substrate and the protein content of larvae fed on it, together with the larval size and the larval protein content. All analyses were conducted using the JMP 10 software (SAS Institute Inc. 2012).

### Ethical approval

This article does not contain any studies with human participants performed by any of the authors. All applicable international, national, and/or institutional guidelines for the care and use of animals were followed.
